# Chinese expert consensus on the treatment of modern combat-related spinal injuries

**DOI:** 10.1186/s40779-019-0196-7

**Published:** 2019-02-20

**Authors:** Zhao-wen Zong, Hao Qin, Si-xu Chen, Jia-zhi Yang, Lei Yang, Lin Zhang, Wen-qiong Du, Xin Zhong, Ren-jie Zhou, Dan Tan, Hao Wu, Representing the Traumatology Branch of the China Medical Rescue Association, the PLA Professional Committee and, the Youth Committee on Disaster Medicine, and, the Disaster Medicine Branch of the Chongqing Association of Integrative Medicine

**Affiliations:** 1 State Key Laboratory of Trauma, Burn and Combined Injury, Department of War Wound Rescue Skills Training, Base of Army Health Service Training, Army Medical University, Chongqing, 400038 China; 2 Department of Tactical Health Service, NCO School of Army Medical University, Shijiazhuang, 050000 China; 3 Xinqiao Hospital, Army Medical University, Chongqing, 400037 China

## Abstract

The battlefield treatments of spinal and spinal cord injury vary from civilian settings. However, there is no unified battlefield treatment guidelines for spine trauma in PLA. An expert consensus is reached, based on spine trauma epidemiology and the concepts of battlefield treatment combined with the existing levels of military medical care in modern warfare. Since the specialized treatment for spine trauma are no significant difference between civilian settings and modern war, the first aid, emergency treatment and early treatment of spine trauma are introduced separately in three levels in this consensus. In Level I facilities, the fast and accurate evaluation of spine trauma followed by fixation and stabilization are recommended during the first-aid stage. Re-evaluation, further treatment for possible hemorrhagic shock, dyspnea and infection are recommended at Level II facilities. At Level III facilities, it is recommended to strengthen the intensive care and the prevention of urinary system and lung infection for the wounded with severe spinal injury, however, spinal surgery is not recommended in a battlefield hospital. The grading standard for evidence evaluation and recommendation was used to reach this expert consensus.

## Main text

Spinal fracture is a common traumatic injury, accounting for approximately 4.3% of all bone fractures. Approximately 10% of spinal fractures are associated with spinal cord injury. In previous wars, including the Vietnam War, the incidence of spinal injury was approximately 1%. However, in Operation Iraqi Freedom (OIF) and Operation Enduring Freedom (OEF), the incidence of combat-related spine and spinal cord injuries ranged from 5.45–11.00%, showing an increasing trend. The management of spine and spinal cord injuries during combat differs significantly from that during peacetime. The care received by patients with spine and spinal cord injuries in civilian medical centers mainly relies on the capacity of medical personnel in the emergency medical care system. Patients are first fixed with spinal fixation devices and are then transported to trauma centers for definitive treatment. Under normal circumstances, this transportation duration does not exceed 1 h. In contrast, multiple steps are involved in the care of patients with combat injuries between the location of the injury and the local medical center, and the unpredictable weather conditions and changes in the enemy’s states make the care for these patients extremely complicated. Based on the epidemiology of spinal injuries in modern wars, this expert consensus was developed according to the treatment needs for combat-related spinal injuries and in combination with the current treatment steps used in the Chinese military. In the soon-to-be-issued *“Guidelines for treating war injuries”*, the current medical care scheme will be adjusted, dividing the contents of the current care in the emergency treatment phase into two phases, including first aid on the scene and early-phase medical care. Upon release of these rules, this consensus will be adjusted according to the latest version of the combat injury treatment rules.

The levels of evidence and recommendations in the process of compiling this expert consensus are mainly based on criteria recommended by the Oxford Centre for Evidence-based Medicine (OCEBM) and some common standards in the clinical medical research [1–3]. Due to the specificity of the care for combat-related injuries (for example, the inability to carry out randomized double-blind studies), we used the evidence quality grading and recommendation strength system of the Grades of Recommendation, Assessment, Development, and Evaluation (GRADE) [4]. The advantage of the GRADE system is that a strong recommendation can be proposed according to a comprehensive assessment under circumstances of low levels of evidence and the absence of high-level evidence-based medicine. Therefore, this GRAGE system is appropriate for combat-related injury assessment and recommendation grading of the diagnostic method. The evidence level and recommendation class are provided for each opinion in this article, which are presented as the evidence level/recommendation class.

**Consensus 1:** Compared with daily life and previous wars, the incidence of spinal injury is high in modern warfare, along with an increase in the incidence of penetrating spinal fractures, multilevel noncontiguous spinal fractures, associated injuries and associated spinal cord injuries. To save more lives and prevent secondary damage of the nerves, it is necessary to strengthen the treatment of spinal injuries during each medical care step based on the characteristics of these injuries **(Level C/ Class I)**.

## Overview

Unlike daily life and early wars such as the Vietnam War, explosive devices in modern wars have become the main cause of spinal injuries [5–7], resulting in a casualty rate higher than that associated with bullet wounds, leading to the characteristics of modern combat-related spinal injuries that are distinct from the injuries encountered in civilian life and previous wars. These characteristics mainly include the following. 1) The incidence of combat-related spinal injuries significantly increased, reaching as high as 5.45–11.10%, which was substantially higher than the rate of 1.00% in previous wars [5–7]. The main cause for this change is that the increased killing effects of weapons in modern warfare lead to the increased probability of spinal injury. In the meantime, spinal injuries were seriously underestimated in previous wars due to outdated diagnostic methods [7]. 2) There were more associated injuries. In OIF/OEF, 78% of patients with spinal fractures had at least one associated injury, and the average number of associated injury locations was 3.4. These associated injuries were mostly severe thoracoabdominal injuries, traumatic brain injuries and traumatic amputations [8]. Under these circumstances, there may be multiple life-threatening conditions that required medical care following high-level traumatic life support rules and could not be limited to the treatment of spinal injury alone. 3) Seventy-six percent of the injuries were multilevel noncontiguous spinal fractures, and the usual incidence of these injuries is only 10–15% [8]. 4) The mechanisms of blast injuries lead to an increased incidence of burst fractures of the lower lumbar vertebra and lumbosacral separation, which required higher levels of wartime medical care [9–11]. 5) The incidence of penetrating spinal injuries is much higher than usual, and the ratio of these injuries requiring surgical interventions in field hospitals was higher than that of closed injuries [12]. 6) The incidence of associated spinal cord injuries is approximately 18%, which is higher than the 10% rate in nonbattle injuries [12, 13]. In the meantime, neurological functions recover poorly following combat-related injuries, requiring long-term, costly rehabilitation treatment [14, 15]. In summary, the incidence of spinal injuries is high, and the incidence of penetrating spinal fractures, noncontiguous spinal fractures, associated injuries and associated spinal cord injuries is increased in modern wars, resulting in different requirements for first aid on the scene, surgical treatment in the field and subsequent rehabilitation.

**Consensus 2:** During the first-aid phase at the battle scene, the “MARCH (massive hemorrhage, airway, respiration, circulation and hypothermia) sequential method” or the simple triage and rapid treatment (START) is recommended to rapidly assess and treat lethal injuries, including massive hemorrhage, followed by a comprehensive determination of whether there are spinal cord injuries requiring immobilization at the scene using methods of injury mechanism determination and rapid and simple examinations **(Level B/Class I)**.

**Consensus 3:** For patients with penetrating injuries, it is not recommended to stabilize the cervical vertebrae using a cervical collar at the battle scene unless the ballistics information suggests that the cervical vertebrae may be injured **(Level C/Class III)**.

**Consensus 4:** The cervical vertebrae should be stabilized with a cervical collar at the battle scene for patients with neurological function impairment, cervical pain or deformity following blast or other blunt injuries **(Level B/Class IIa)**.

**Consensus 5:** The long axis of the body should be kept in a straight line during the movement and transportation of patients with spine and spinal cord injuries to prevent the movement or twisting of the entire spine, which may cause additional damage to the spine and spinal cord **(Level B/Class IIa)**.

**Consensus 6:** Patients with open spinal fractures should take preventive oral antibiotics. It is recommended to take 400 mg moxifloxacin or 500 mg levofloxacin orally, and the wounds should be wrapped with sterile dressings **(Level B/Class IIa)**.

**Consensus 7:** When a patient with a spine and spinal cord injury is evacuated, any equipment on the patient and hard objects in the pockets should be removed to prevent pressure sores **(Level B/Class IIa)**.

## First aid of combat-related spinal injury at the battle scene

First aid at the battle scene is usually completed by battalion and lower-level health agencies or equivalent agencies within 10 min following an injury. The key to the medical care of combat-related spine injury at the battle scene is to rapidly assess the patient according to the rules of high-level traumatic life support and rapidly identify the conditions that threaten the patient’s life, including massive hemorrhage, hemorrhagic shock, airway obstruction and tension pneumothorax. At the time of rapid treatment, the spine should also be stabilized using rapid and effective methods, and the patient should be evacuated promptly.

### Injury assessment at the battle scene

The primary premise of the injury assessment at the battle scene is the safety of the battlefield environment. Injury assessment should be performed in the absence of enemy fire threats or in the presence of effective shelter protection. The injury assessment at the battle scene should be done promptly and accurately, meaning that life-threatening conditions should be identified and treated as fast as possible. The MARCH sequential method or the START method is recommended for prompt injury assessment to sequentially assess whether the injured person has life-threatening massive hemorrhage, airway obstruction, tension pneumothorax or open pneumothorax, impaired circulation or hypothermia. The life-threatening conditions should be treated simultaneously during the injury assessment, and the presence or absence of spinal and neural functional injuries should then be assessed [16, 17].

The spine and spinal cord should be examined using simple and rapid methods at the battle scene to determine whether there are spine and spinal cord injuries requiring stabilization. The key points of examination include the following [18]. 1) The spine may be quickly examined in a top-down manner according to the patient’s main complaint. During the examination, the patient should not sit up or bend the spine forward but instead should only be turned at the site. Generally, the patient needs to be examined by turning the body along the axis to prevent aggravation of spine and spinal cord injuries. The main items to be examined include whether there is local pressure pain, skin contusion or local spine deformity. 2) The neurological functions should be assessed using simple methods, such as asking the patient to lift the arms and legs, and the functional limitations should be recorded. The patient is then examined from the shoulder to the feet to determine whether there is sensory loss. A comprehensive neurological functional examination does not need to be performed at the battle scene, as it does not provide much significance to the treatment and takes time. 3) Understanding the injury mechanism is beneficial for the determination of whether the patient requires external fixation to stabilize the spine. For example, it is highly possible that the spines of patients with blunt spinal cord injuries may be unstable and require stabilization with external fixation. However, patients with penetrating injuries normally have stable spines and under most circumstances do not need spine stabilization at the battle scene.

### Fixation and stabilization of the injured spine

Currently, advanced trauma life support procedures suggest that all patients with injuries above the collarbone or head injuries accompanied with coma should be fixed with a semi-rigid cervical collar and hardback plate [19, 20]. However, this practice is not practical at the battlefield, as the provision of correct neck support and spine fixation may expose multiple medical personnel to enemy fire, which may lead to more casualties. In the meantime, external fixations such as the neck collar may increase cervical pressure, affecting venous return and compressing the airway, which is more pronounced with associated cervical injuries and prohibits treatment of the airway and other cervical injuries [21]. There is still some controversy regarding the use of external fixation devices, such as neck collars, to immobilize the cervical spine. Currently, the relatively unified views are as follows. 1) Spinal immobilization is not recommended for patients with penetrating trauma unless the ballistics assessment indicates direct cervical injuries [22]. Unlike blunt injuries, penetrating injuries rarely cause destabilization of the spine [23, 24]. Arishita et al. [25]. retrospectively analyzed the conditions of patients with penetrating cervical cord injuries in the Vietnam War and found that the majority of these injuries were fatal and caused immediate death, and only 1.4% of all casualties benefited from cervical collar immobilization. Immobilization during the first-aid phase at the battle scene might have increased the casualty rate of medical personnel by 10%. In OIF/OEF, among the 90 British military casualties with a penetrating neck injury, the incidence of cervical spine fracture or cervical spinal cord injury was 22%, and only 6 patients survived the trip to the hospital. Among the 6 survivors, 4 died in the hospital within 72 h, and only 1 survived to reach a surgical facility [26]. Therefore, Ramasamy et al. [27] believed that penetrating injuries were associated with a high mortality rate and that the penetrating injuries were unlikely to cause unstable cervical spine fractures. The risk/benefit ratio of mandatory fixation with cervical collars at the battle scene in patients with penetrating injuries is low. 2) In the presence of neurological function impairment or local pain, proper measures should be taken to stabilize the spine in patients with blunt injuries. Detailed symptoms include the following: A. changes in consciousness or a Glasgow coma score < 15; B. local pain or tenderness in the spine; C. neurological dysfunctions, including bilateral paralysis, partial paralysis, numbness, stabbing pain, and neurogenic shock below the injury site; and D. observation of spine deformity during the examination at the battle scene. The incidence of blast injury combined with spine fractures is approximately 8%. Therefore, the clinical practical guidelines of the Tactical Combat Casualty Care suggest that cervical collars should be used in patients with blast injuries at the battle scene [20]. Taddeo et al. [28] analyzed the use of cervical collars in patients with cervical spine injuries following improvised explosive device (IED) trauma between 2008 and 2011 and found that dismounted IED trauma only occurred in 326 patients among the 15,693 wounded. The rate of patients with prehospital cervical collar placement was 7.6%. Nineteen patients (5.8%) had cervical fractures, and only 4 patients (1.2%) had an unstable spine confirmed by radiographic imaging. None of the 19 IED patients had received prehospital cervical collar placement. The authors believed that a dismounted IED was associated with a low risk of cervical spine injury and suggested that cervical collar placement was not required for patients who were alert, had sat up, walked or could spontaneously move their necks [23].

Of the spinal immobilization techniques, the standard emergency equipment that is easy to operate, such as rigid cervical collars, standard fixation stretchers or vacuum spine boards, is recommended [29]. In the absence of special equipment, the spine should be temporarily immobilized with materials such as a rigid backboard, door panel or bed board that can keep the thoracolumbar spine stable. During the spinal immobilization, the entire load-bearing spine should be fixed and maintained as a whole to achieve a proper immobilization. The supine position is the most stable position that can not only ensure subsequent support during the treatment and transportation of the injured individual but also provide the optimal condition for additional examinations, necessary resuscitation and treatments.

### Transportation of patients with spine and spinal cord injuries

During the movement and transportation of patients with spine and spinal cord injuries, it is crucial to avoid movement or twisting between the spinal segments. The patient should be transported and evacuated on a rigid backboard after fixing the head, neck and body. The patient should be kept on the backboard during the evacuation. If it is necessary to move the patient, multiple people should be involved in moving the patient without bending the spine. A rigid stretcher should be used to prevent excessive extension or bending of the spine [30, 31].

### Evacuation

The patient should be transported and evacuated on a rigid backboard and be kept on the backboard during the evacuation. If it is required to move the patient, multiple people should be involved in moving the patient without bending the spine. During the evacuation, the devices on the patient and hard objects in the pockets should be removed to prevent pressure sores.

### Other treatments

To reduce the probability of infection in patients with open spinal injuries, it is recommended to wrap the wound with a sterile dressing, and patients with open spine fractures at the tactical zone should take preventive oral antibiotics. It is recommended to take 400 mg moxifloxacin or 500 mg levofloxacin orally [23, 32].

**Consensus 8:** According to the emergency treatment agencies, the key to the treatment of combat-related spinal injury is to prevent shock, respiratory circulatory failure and lung infection and further improve spine stability **(Level B/Class I)**.

**Consensus 9:** When a patient with a combat-related spinal injury has many other injuries, he or she is more prone to hemorrhagic shock and should be subjected to active anti-shock treatments. It is recommended to infuse the patient with red blood cells/fresh frozen plasma/platelets in a 1:1:1 ratio for fluid resuscitation in the first place **(Level A/Class I)**.

**Consensus 10:** A patient is diagnosed with neurogenic shock in the presence of flaccid paralysis, moderate hypotension and varying degrees of bradycardia after spinal fracture. Under this circumstance, the patient can be treated with vasoactive drugs to increase blood pressure **(Level A/Class IIa)**.

**Consensus 11:** When a patient with a cervical spinal cord injury has difficulty breathing, tracheal intubation or cricothyroid laryngotomy should be performed, and an esophageal balloon should be used for assisted ventilation **(Level B/Class I)**.

## Emergency treatment of combat-related spine injuries

Emergency treatment is normally completed by the regiment or brigade ambulance service or equivalent medical agencies within 3 h of the injury. At the emergency medical agencies, such as company or battalion clinics, the patient should undergo a second injury assessment to prevent the missed diagnosis of life-threatening injuries. In the meantime, an in-depth assessment should be performed for spine and spinal cord injuries. The emergency treatment is mainly symptomatic treatment to prevent early-phase complications, including respiratory circulatory failure and pulmonary infection.

### Shock prevention

When combined with many other injuries, patients with combat-related spinal injuries are more prone to hemorrhagic shock and should receive active anti-shock treatments. The Chinese military has prepared blood products at this level of medical agencies. Patients with hemorrhagic shock can be resuscitated with a combination of blood products and crystalloid solution as well as a colloid solution. It is recommended to infuse the patient with red blood cells/fresh frozen plasma/platelets in a 1:1:1 ratio in the first place [24, 26]. When there is not a sufficient blood product supply, fresh whole blood can be collected and transfused to the wounded [33]. Freeze-dried plasma can be stored at 2–35°C for 15–24 months with 75–100% of its clotting activity retained. Currently, LyoPlas and LyoPhil are the commercially available products. If blood products, such as red blood cells and fresh frozen plasma or whole blood, are unavailable, freeze-dried plasma can be used for resuscitation. Currently, freeze-dried plasma has been approved for use in the British, French, German and Israel military [34], but it has not been approved by the American Food and Drug Administration. Only some US special forces are allowed to carry French freeze-dried plasma. When blood products, such as red blood cells and fresh frozen plasma, whole blood or freeze-dried plasma, are unavailable, hydroxyethyl starch can be used for fluid resuscitation [35].

Neurogenic shock can also occur during combat-related spinal injury, and the associated clinical features and medical treatments are different from those of hemorrhagic shock. The mechanism of neurogenic shock is that spinal cord injury leads to interruption of the sympathetic nerve innervated by thoracic segment 1 to lumber segment 2, resulting in weakened inhibition of the vagus nerve and, subsequently, the presence of moderate hypotension and bradycardia. Neurogenic shock is determined by the presence of flaccid paralysis, moderate hypotension and varying degrees of bradycardia after spinal fracture. Under the circumstance of neurogenic shock, blood pressure can be increased by vasoactive drugs [36, 37].

### Respiratory support

When a spinal cord injury is accompanied by difficulty in breathing, tracheal intubation or cricothyroid laryngotomy should be performed, and an esophageal balloon should be used for assisted ventilation. The airway should also be cleared. It is critical to maintain a sufficient oxygen supply for the spinal cord, which often requires an auxiliary breathing apparatus and oxygen saturation monitors. Similarly, it is crucial to prevent failures in the airway, respiratory and circulatory system to ensure adequate recovery of the injured spinal cord [38, 39].

### Catheterization

The indwelling catheter should be placed using aseptic techniques in patients with urinary retention. The external end of the catheter should be wrapped with a sterile cloth, and the urine should be released once every 4–6 h. Patients should also take oral urinary-sensitive antibiotics, such as 100 mg furan-pyrimidine 3 times per day.

### Infection prevention

Antibacterial medications such as penicillin and gentamicin should be used. Tetanus antitoxin serum should be administered to patients with open injuries.

### Immobilization of patients with suspected spine and spinal cord injuries

Spine immobilization is required at the emergency medical agency for patients with suspected spine and spinal cord injuries. One effective cervical spine immobilization method is to use a sandbag or a towel roll to fix the neck on both sides, tape the patient’s forehead to the spinal hardboard and use a rigid cervical collar to prevent cervical overextension. A simple soft, rescuing, rigid or Philadelphia cervical collar is insufficient for immobilization.

Flat brackets or sternal-occipital-mandibular braces are not practical for the battlefield. Fixing the patient on a standard long spine hardboard is a standard method for immobilization of the thoracolumbar spine. This method provides the maximum support for the thoracolumbar spine when turning the patient over (for example, when the airway needs to be cleared or the patient needs to vomit).

However, axial traction and hardboard fixation may still cause spinal translation and rotation. Therefore, rotational activities should be minimized in patients with suspected spinal injuries. Spinal immobilization devices can be removed only when spine instability is excluded by radiography and clinical assessments.

### Evacuation

If conditions permit, the injured person should be evacuated as soon as possible. For patients with an associated spinal cord injury, pressure sores should be prevented during the evacuation. The pressured positions should be padded with soft pillows, balloons or foam. The injured person should be turned regularly by fixing him/her to the middle of a stretcher bed. Heatstroke and freezing should also be prevented. However, hot-water bags should not be used to prevent scalding the patient.

**Consensus 12:** In early-phase medical agencies, the key to the treatment of patients with combat-related spinal injuries is further injury assessment. Based on the assessment, it is required to strengthen the intensive care and treat patients with urinary system and lung infections, shock, pressure sores, deep vein thrombosis and disturbances of water, electrolyte and acid-base balances. Patients with indications should be subjected to surgical treatments. After being stabilized, the patients should be transported to specialized treatment agencies as soon as possible **(Level B/Class III)**.

**Consensus 13:** In early-phase medical agencies, patients should be subjected to a second detailed injury assessment to prevent the missed diagnosis of life-threatening injuries. In the meantime, the spine and spinal cord injuries should also be assessed carefully, and the nerve injury status should be recorded accurately, which is critical for the assessment of disease progression and selection of treatment plans for the injured patient **(Level B/Class I)**.

**Consensus 14:** The airway management should be strengthened for patients with associated cervical spinal cord injuries. A ventilator should be used in the presence of a respiratory failure in patients with high-level cervical spinal cord injury to maintain normal oxygenation **(Level B/Class I)**.

**Consensus 15:** Patients with spinal cord injuries should be subjected to active fluid resuscitation such that the mean arterial pressure is maintained at 80–90 mmHg **(Level B/Class I)**.

**Consensus 16:** Patients with spinal cord injuries have an increased risk of deep vein thrombosis. Physical therapy includes massage and ankle exercises. Low-molecular-weight heparin is recommended for drug treatment **(Level B/Class IIa)**.

**Consensus 17:** It is recommended to use a Foley catheter during treatment at early-phase medical agencies, and the urine should be released once every 4–6 h to prevent infection of the urinary system **(Level A/Class I)**.

**Consensus 18:** Corticosteroids are not recommended for the treatment of spinal cord injuries in battlefield hospitals **(Level B/Class III)**.

**Consensus 19:** It is important to prevent and treat disturbances of water, electrolyte and acid-base balances in battlefield hospitals, especially hyponatremia **(Level B/Class IIa)**.

**Consensus 20:** Due to the lack of specialized physicians, susceptibility to infection and a significant increase in the incidence of complications, it is normally not recommended to perform spine surgery in battlefield hospitals **(Level B/Class III)**.

**Consensus 21:** The main indications for spine surgery in battlefield hospitals include the following: (1) incomplete paralysis with progressive neurological functional deterioration; (2) penetrating spine fracture in the presence of cerebrospinal fluid leakage or with associated thoracic and abdominal organ injuries; (3) patients with incomplete paralysis whose evacuation may be delayed for > 5 d; and (4) patients with predicted aggravation of their spinal cord injury **(Level B/Class IIa)**.

## Early-phase treatments of combat-related spine injuries

The early-phase treatments of combat-related spine injuries are normally completed at division aid stations or equivalent medical agencies at approximately 6 h after injury. In early-phase medical agencies, the key to the treatment of patients with combat-related spinal injuries is further injury assessment. Based on the assessment, it is required to strengthen the intensive care and treat patients with urinary system and lung infections, shock, pressure sores, deep vein thrombosis and disturbances of water, electrolyte and acid-base balances. Patients with indications should receive surgical treatments. The patients should be transported to specialized treatment agencies as soon as possible once they are stable.

### Further assessment of the injured

In the early-phase medical agencies such as division field medical centers, patients should be subjected to a second detailed injury assessment to prevent the missed diagnosis of life-threatening injuries. In the meantime, the spine and spinal cord injuries should also be assessed carefully, and the nerve injury status should be recorded accurately, which is critical for the assessment of disease progression and selection of treatment plans for the injured patient.

A thorough second assessment includes a detailed examination of the entire spine and neurological functions. With the protection of cervical collar or axial fixation, the entire spine of a patient is examined by turning the body. The back of the patients should be examined to determine whether there are any traumas, deformities or congestions. The spine should be palpated to determine whether there is misalignment or any increase in the interspinal spaces. The location of a lacerated wound or bruise on the head can help determine the mechanism of cervical spinal injury, with a laceration at the occipital region indicating overbending injury and injuries at the forehead or above indicating an overextension injury or axial compression injury. Identification of a spinal injury in one position does not indicate the absence of spinal injuries at other positions. Normally, 8–28% of patients with spinal cord injuries have spinal trauma in other noncontiguous locations, and this ratio can be as high as 76% at wartime, with 30% of these types of injuries remaining undetected at the first assessment.

Normally, the American Spinal Injury Association recommends examination of the nervous system, including motor, sensory, reflex and sphincter functions, to determine the severity and location of the injury [40]. In particular, dynamic and multiple assessments are required to determine the presence of progressive deterioration of neurological functions. Under the circumstance of neurological function deterioration, emergent decompression and spine immobilization are required.

X-ray devices are available in early-phase medical agencies of the Chinese military. Patients with combat-related spinal injuries who are stable should undergo X-ray examination to determine the types of spinal injury to guide treatment.

### Respiratory support and prevention of lung infection

For cervical spinal cord-injured patients with severe respiratory function damage who have been established to have a definitive airway by the emergency medical agencies, strengthened management of the airway should be continued. Mechanical ventilation should be used when necessary, and the oxygen saturation should be maintained at ≥90% [41, 42].

Functional impairment in the respiratory system is the most common complication and cause of death within weeks in patients with spine and spinal cord injuries, and it can cause other complications, including the inability to cough, lung infection, pulmonary atelectasis and respiratory failure. The main treatments are as follows: 1) when high-level cervical spinal cord injuries affect the lower respiratory center, patients may have trouble breathing and require tracheal intubation or tracheal incision, and a ventilator is needed for breathing support; 2) patients should be turned over and patted on the back to promote sputum excretion; 3) use of drugs that inhibit the parasympathetic nervous system; 4) effective antibiotics; and 5) effective medication for sputum excretion and atomization.

### Circulatory support

Sufficient perfusion pressure is critical in the prevention of secondary injury following spinal cord injury. It is generally believed that the mean arterial pressure should be maintained at approximately 80–90 mmHg, which can be achieved in patients with non-neurogenic shock by sufficient fluid resuscitation. However, improving the low blood volume in patients with neurogenic shock by simply using an appropriate crystalloid solution and blood or blood products is not sufficient to maintain the required mean arterial pressure. Normally, vasopressors that contain β1 agonists, including dopamine and dobutamine, are needed to ensure that the mean arterial pressure is maintained at approximately 80–90 mmHg [37, 43].

The function of the parasympathetic nervous system is disrupted following cervical and thoracic spinal cord injury, which may cause cardiac arrhythmia. Patients with severe bradycardia respond well to atropine. Hypovolemic shock, but not neurogenic shock, is highly suspected if patients have tachycardia and associated hypotension. However, neurogenic shock is highly suspected if patients have bradycardia and associated hypotension.

### Prevention of deep vein thrombosis

Due to the absence of normal muscle activities in the body, blood flows slowly in paraplegic patients. As a result, paraplegic patients are prone to deep vein thrombosis. Shedding of the emboli may cause acute pulmonary embolism, which is a leading cause of death in patients with spinal cord injury [44]. When caring for these patients, special attention should be paid to the passive activities of the limbs, which should be pressed in a from-far-to-close rhythm to promote blood flow and prevent thrombosis. The results from randomized double-blind studies showed that, among the age- and gender-matched patients at 1 month after injury, the risk of death from pulmonary embolism in patients with spinal cord injury increased 500-fold [44, 45].

The main measures to prevent thrombosis include physical methods and drug treatments. Physical methods include massage and ankle pump exercises, and the effects of low-molecular-weight heparin are best among the drug treatments. Rivaroxaban can also be used, and this drug has relatively minor side effects. It is generally believed that preventive drugs should be used until 30 d after the spinal cord injury. An inferior vena cava filter can be placed in patients with contraindications against anticoagulant drugs, and this needs to be performed at specialized medical agencies.

### Prevention of urinary system infection

Changes in detrusor function, bladder sensitivity and reduction of sphincter function have a negative impact on bladder emptying. These factors also increase the infection rate of the urinary system and contribute to the formation of bladder and kidney stones. To reduce the incidence of these complications, the Foley catheter is recommended during treatment at early-phase medical agencies, and the urine should be released once every 4–6 h to prevent infection of the urinary system. Preventive antibiotics are not recommended. However, any suspicious urinary tract infections should be treated [46].

### Use of corticosteroids

Corticosteroids are the most studied neuroprotective drug. Currently, in multicentered controlled clinical trials, high-dose methylprednisolone is the only corticosteroid used as a neuroprotective agent. However, some recent studies have questioned the effects of methylprednisolone on acute spinal cord injury. While some retrospective studies questioned the effects of methylprednisolone, more studies showed that the incidence of complications increased among patients treated with methylprednisolone, including a high risk of pulmonary infection, long hospital stay, pulmonary embolism, wound infection, gastrointestinal hemorrhage and sepsis. Based on these findings, the spinal cord injury clinical treatment guidelines emphasized that “24-hour or 48-hour infusions of methylprednisolone is an option for the treatment of patients with acute spinal cord injury. However, this is only applicable to the understanding of adverse effects and that it has high clinical benefits” [47]. The US military Tactical Combat Casualty Care guidelines suggest that corticosteroids should be strictly prohibited for patients with open spine and spinal cord injuries and are not recommended for patients with blunt spine and spinal cord injuries [48].

### Prevention of disturbances of water, electrolyte and acid-base balances

Hyponatremia is a common complication of early-phase spinal cord injury, with an incidence of 13–19%. The mechanism of hyponatremia is still unclear. It is generally believed that following spinal cord injury, the sensory conduction pathway is interrupted and the special channel for the regulation of renal function is blocked, which continuously inhibits the secretion of antidiuretic hormone, resulting in polyuria. The use of a large amount of diuretics in the early stage of the injury increases water and electrolyte (mainly Na^+^) secretion. Fluid is supplemented to maintain the balance of water and electrolytes, which dilutes the blood and leads to hyponatremia. However, an increased body fluid volume leads to continuous Na^+^ secretion in the urine. If patients cannot eat at the early stage, hyponatremia can be caused by several factors, including insufficient intravenous Na^+^ [43].

The current clinical experience is that the use of isotonic fluid for rehydration in the early stage of cervical spinal cord injury prior to the presence of hyponatremia may delay its occurrence.

### Fixation

If surgery is not planned for patients with unstable spine fractures, the patients can be evacuated after spine immobilization with cervical collars, Halo braces, and head, cervical, thoracic and lumbar braces [49]. Among these fixations, the Halo brace is commonly used by the following method. A Halo brace of an appropriate size is selected based on the head diameter, placed at 1 cm above the eyebrow and encircling the head. The Halo brace is temporally fixed using a plastic stick. After closing the eyelids, the skin is sterilized and local anesthesia is administered. The needle is screwed in through the hole on the brace in the reverse direction to 8 in.-pound in adults and 4 in.-pound in children < 8 years old. The needle should be tightened within 24 h. The Halo brace can be used if there is a plan to use it for definitive treatment or when cervical spine traction cannot be completed using Gardner-Wells tongs. When the patient is transferred to the sickbed, placing a Halo vest under the patient can facilitate fixation of the brace on the vest during spine traction. The open Halo brace is superior to the entire brace structure of the previous Halo brace since the open brace can be applied without needing to place the patient’s head on the traction frame.

### Early-phase surgical treatments of patients with spine and spinal cord injuries

Due to the lack of specialized physicians and susceptibility to infection, the incidence of complications is significantly increased if spine surgery is performed in battlefield hospitals [50–52]. Schoenfeld et al. [53] compared neurological function recovery and complications in patients who underwent spine surgery in battlefield hospitals and level IV medical agencies during 2010–2011. A total of 30 patients underwent spine surgery in battlefield hospitals, and 20 patients underwent spine surgery in the Landstuhl Regional Medical Center. The incidence of complications among patients undergoing spine surgery in battlefield hospitals was 40%, and 23 patients required a second surgery. However, the incidence of complications among patients undergoing spine surgery in the Landstuhl Regional Medical Center was only 20%. No significant difference was observed between the two groups in the recovery of neurological functions. The authors believed that spine surgery in the battlefield hospitals was not beneficial for the recovery of neurological functions but instead increased the incidence of complications, including the need for a second surgery. Therefore, under general circumstances, it is not recommended to perform spine surgery in battlefield hospitals [51, 54].

In the presence of the following circumstances, whether or not to perform surgery can be decided based on the availability of specialized physicians and equipment:

1. Incomplete paralysis with progressive neurological functional deterioration. Nerve injuries following spine fractures include complete and incomplete paralysis with neurological symptom deterioration, incomplete paralysis with no deterioration in neurological symptoms, and normal neurological functions. For patients with complete paralysis, the performance of surgery and early surgery have little effect on the recovery of neurological functions. For patients with incomplete paralysis and no deterioration in neurological functions or patients with normal neurological functions, the need for spine stabilizing surgery can be determined based on spine stability when the patients are evacuated to specialized hospitals. Acute decompression at the battle scene is required for patients with incomplete paralysis and deterioration of neurological symptoms.
2. Penetrating spine and spinal cord injuries. In modern wars, the incidence of penetrating injury-induced spine and spinal cord injuries has increased along with the widespread use of high-speed light weapons. Generally, simple debridement is needed for penetrating spine and spinal cord injuries, but thorough intraspinal canal debridement and spinal cord decompression are not recommended, as they are not beneficial for the recovery of neurological functions but instead increase the risk of complications including infection, cerebrospinal fluid leakage and hemorrhage [55, 56]. Surgery is required when penetrating spinal fractures are associated with compression-induced incomplete and deteriorating spinal cord or cauda equina injuries or when there is cerebrospinal fluid leakage or associated thoracic abdominal organ injuries [57–60].

Penetrating spine and spinal cord injuries are often associated with cervical and thoracic abdominal tissue and organ injuries, which should be treated with high priority. Thoracic drainage is generally required when there is associated pulmonary contusion. Laparotomy is required in the presence of abdominal organ injuries. Under most circumstances, when there are associated abdominal organ injuries, the initial point of injury is on the back, followed by entry into the abdomen through the spine, and laparotomy can generally be performed first. The fascia can be used for closure if there is cerebrospinal fluid leakage, followed by simple posterior debridement. Patients with penetrating spine injuries have a high risk of infection and should take preventive antibiotics to prevent infection [61]. Patients with penetrating spinal injuries but no hollow viscus injuries are recommended to take preventive antibiotics for 3–5 d. Normally, intravenous injection of cephazolin at 1 g/time/8 h is recommended. Under the circumstance of continuous cerebrospinal fluid leakage caused by broken spinal dura mater, intravenous injection of ceftriaxone sodium at 1 g/time/12 h is recommended if there is suspected meningitis [55]. Associated hollow viscus injury should be considered in patients with penetrating projection spinal injuries. If a projection injury runs through the pharynx, esophagus or colon, additional precautions are required to prevent spinal infection. This is especially important if the bullet first penetrates the hollow viscus and then the spine, but it is not clinically significant if the bullet first penetrates the spine and then the hollow viscus.

(3) Patients whose evacuation may be delayed for more than 5 d. This is especially applicable for patients with incomplete paralysis, as surgery can facilitate recovery of neurological functions. However, surgery has little effect on patients with complete paralysis or normal neurological functions [16, 62].
(4) Predicted aggravation of spinal cord injury during the evacuation. Experienced spine surgeons are first needed to determine whether the evacuation may aggravate the patient’s spinal cord injury and whether to perform spine stabilization surgery in an early-phase medical agency [16, 62, 63].

In early-phase medical agencies, the key to the treatment of patients with combat-related spinal injuries is an extensive injury assessment of the patients. Based on the assessment, it is required to strengthen the intensive care and treat patients with urinary system and lung infections, shock, pressure sores, deep vein thrombosis and disturbances of water, electrolyte and acid-base balances. Patients with indications should receive surgical treatments. After being stabilized, patients should be transported to specialized medical agencies as soon as possible.

## Abbreviations

ATLS: Advanced trauma life support
IED: Improvised explosive device
MARCH: Massive hemorrhage, airway, respiration, circulation and hypothermia
NCO: Non-commissioned officer
OCEBM: Oxford Centre for Evidence-based Medicine
OEF: Operation Iraqi Freedom
OIF: Operation Enduring Freedom
PLA: Chinese people’s liberation army
START: Simple triage and rapid treatment
